# Correction: The small and large ribosomal subunits depend on each other for stability and accumulation

**DOI:** 10.26508/lsa.201900508

**Published:** 2019-08-01

**Authors:** Brian Gregory, Nusrat Rahman, Ananth Bommakanti, Md Shamsuzzaman, Mamata Thapa, Alana Lescure, Janice M Zengel, Lasse Lindahl

**Affiliations:** Department of Biological Sciences, University of Maryland, Baltimore, MD, USA

See original article: The small and large ribosomal subunits depend on each other for stability and accumulation, 2(2), 2019.

Following publication, the authors noted that Table S1 was missing from the published version of the article, a mistake introduced during production. We apologize to our readers for this error. The HTML version of the article has been corrected and the table is given below.

Table S1 Strains used.

