# Supplementary material for: The small and large ribosomal subunits depend on each other for stability and accumulation
Source: Life Sci Alliance. 2019 Mar 5;2(2):e201800150. doi: 10.26508/lsa.201800150 (PMC6402506; doi:10.26508/lsa.201800150)
Supplement: Supplementary file 5 [file LSA-2018-00150_TableS1.pdf]

Table S1. Strains used

Strain name	Gene expressed from GAL promoter	Protein expressed from GAL promoter	Chromosomal genotype	Plasmid	Reference
BY4741	None	None	Matα; his3Δ1; leu2Δ0; lys2Δ0; ura3Δ0	None	Ferreira-Cerca *et al.*, 2005
Y699	*RPS5*	uS7/S5	his3Δ1; leu2Δ0; lys2Δ0; ura3Δ0; YJR123w::KanMX4	Ycplac111 Pgal-RPS5	Ferreira-Cerca *et al.*, 2005
Y259	*RPS9A*	uS4/S9	his3Δ1; leu2Δ0; ura3Δ0;YBR189w::KanMX4; YPL081w::HIS3	Ycplac111 Pgal-RPS9A	Ferreira-Cerca *et al.*, 2005
Y325	*RPS11A*	uS17/S11	his3Δ1; leu2Δ0; ura3Δ0;YBR048w::KanMX4; YDR025w::HIS3	Ycplac111-pGAL-RPS11A	Ferreira-Cerca *et al.*, 2005
Y399	*RPSS14A*	uS11/S14	his3Δ1; leu2Δ0; ura3Δ0; YJL191w::KanMX4; YCR031c::HIS3MX6	Ycplac111 Pgal-RPS14A	Ferreira-Cerca *et al.*, 2005
Y1198	*RPS20*	uS10/S20	his3Δ1; leu2Δ0; LYS2; met15Δ0; ura3Δ0; YHL015w::kanMX4	Ycplac111 Pgal-RPS20	Ferreira-Cerca *et al.*, 2005
Y336	*RPS31*	eS31/S31	his3Δ1;leu2Δ0;ura3Δ0;MET15;lys2Δ0;YLR167w::KanMX4	Ycplac111 Pgal-RPS31	Ferreira-Cerca *et al.*, 2005
JWY8402	*RPL4A*	uL4/L4	ura3-52/trp1-101/lys2-801/his3-d200/leu2-d1/rpl4b::KANMX6/GAL-3HA-RPL4A (TRP1)	None	Pöll *et al.*, 2009
YLL2083	*RPL4B*	uL4/L4	ade2-101, his3Δ200, ura3-52, lys2 rpl4AΔ::KAN RPL4BΔ::lox pGAL-RPL4, HIS3	pGAL-RPL4B	Thapa et al 2013
Y1092	*RPL5*	uL11/L5	his3-1/leu2-0/ura3-0/YPL131w::KANMX4	Ycplac111 Pgal-RPL5	Pöll *et al.*, 2009
Y1095	*RPL17A*	uL22/L17	his3-1/leu2-0/ura3-0/YKL180w::KANMX4/YJL177w::HIS3MX6	Ycplac111 Pgal-RPL17A	Pöll *et al.*, 2009
Y1101	*RPL40A*	eL40/L40	his3-1/leu2-0/ura3-0/YIL148w::HIS3MX6/YKR094c::KANMX4	Ycplac111 Pgal-RPL40A*	Pöll *et al.*, 2009
Y1103	*RPL43A*	eL43/L43	his3-1/leu2-0/ura3-0/YJR094w-a::HIS3MX6/YPR043w::KANMX4	Ycplac111 Pgal-RPL43A	Pöll *et al.*, 2009
JWY8110	*RPF2*	Rpf2	Matα; trp1; lys2-801; ura3-5;2 his3delta200; pep4::HIS3; prb1Δ1.6R; can; rpf2::GAL-HA3-RPF2 TRP1	None	Zhang et al. 2007
JWY8111	*RRS1*	Rrs1	Matα; trp1; lys2-801; ura3-52; his3delta200; pep4::HIS3; prb1;delra1.6R; can; rrs1::GAL-HA3-RRS1 TRP1	None	Zhang et al. 2007
YJP57	*RRP7*	Rrp7	MATa, ade2, his3, leu2, trp1, ura3, KANMX6-GAL::HA-rrp7, nan1-MYC::HIS3	None	Perez-Fernandez et al. 2007
YLL2500	*uS17/S11; RPL25 expressed from estradiol incucible promoter*	*uS17/S11 expressed from Pgal; uL23-GFP/L25-GFP expressed from estradiol incucible promoter*	his3Δ1; leu2Δ0; ura3Δ0;YBR048w::KanMX4; YDR025w::HIS3; ade2::Z3EV; URA3::PNM3-uL23::GFP	Ycplac111 Pgal RPS17	This paper

* Includes the RpL40 domain only, not the ubiquitin domain
